# Paediatric drug use with focus on *off-label* prescriptions at Swedish hospitals – a nationwide study

**DOI:** 10.1111/j.1651-2227.2012.02656.x

**Published:** 2012-07

**Authors:** E Kimland, P Nydert, V Odlind, Y Böttiger, S Lindemalm

**Affiliations:** 1 Division of Clinical Pharmacology, Karolinska University HospitalStockholm, Sweden; 2 Medical Products AgencyUppsala, Sweden; 3 Astrid Lindgrens Childrens Hospital, Neonatal Unit, Karolinska University HospitalStockholm, Sweden; 4 Division of Clinical Pharmacology, Department of Laboratory Medicine, Karolinska Institutet, Karolinska University HospitalStockholm, Sweden; 5 Department of Clinical Sciences, Intervention and Technology (CLINTEC), Division of Paediatrics, Astrid Lindgrens Children’ s Hospital, Karolinska Institutet, Karolinska University HospitalStockholm, Sweden

**Keywords:** Child, Drug prescription, Hospital care, *Off-label*, Sweden

## Abstract

**Aim:**

To perform a nationwide investigation of paediatric drug use at Swedish hospitals, including an analysis of *off-label* drug use.

**Methods:**

All paediatric hospitals in Sweden were invited to register all prescriptions to children, aged between 0 and 18, during two separate 2-day-periods in 2008. Data were reported and analysed with respect to licence status and proportion of and reasons for *off-label* drug use.

**Results:**

Data on 11 294 prescriptions to 2947 paediatric patients were received. Drugs associated with pain relief, infection, prematurity, nutrition and surgery or anaesthesia were most commonly used. Paracetamol was the most frequently used drug on-label and also among the most commonly used *off-label* drugs. Nearly half (49%) of all administered prescriptions concerned unlicensed drugs, *off-label* drugs or extemporaneously prepared drugs. The corresponding rate among neonates was 69%. Lack of paediatric information in the Summary of Product Characteristics was the main reason for *off-label* classification.

**Conclusions:**

Paediatric *off-label* drug use is common at Swedish hospitals, and nearly half of all prescriptions were not documented for use in children. The findings emphasize a need for paediatric clinical studies as well as compilation of existing clinical experience and scattered evidence, particularly for drug treatment in infants and neonates.

## Introduction

The licensing procedure of new drugs aims at ensuring their safety, efficacy, quality and positive benefit–risk balance. Such an assessment is based on clinical trials, which in the past almost exclusively were performed in adults. As a result, many drugs are neither tested nor authorized for use in children. Consequently, *off-label* drug prescriptions or unlicensed drug use have been reported to occur frequently in the paediatric population (1–5). Thus, use of at least one drug *off-label* or use of unlicensed drugs has been documented in up to 60% of children treated in hospital care in general (1–5) and, in particular, at an even higher rate in neonatal hospital care (4–6).

Although it is well known that many children receive drugs that have not been tested in paediatric patients, no study has shown the magnitude of *off-label* or unlicensed drug use in hospital at a national level. There are several drug utilization studies among children in primary care (4,5), but these cannot be directly translated into paediatric drug use at hospitals. In hospital care, the majority of studies were performed in specialized units or were limited to a certain geographic area.

Lack of paediatric clinical documentation is one important reason for *off-label* drug use. Another critical issue is the absence of appropriate drug formulations for paediatric patients, that is, oral solutions or small enough tablets. Dosing recommendations for children have often been decided by scaling from adult dosage, although available methods for scaling do not give sufficiently adequate estimates (7).

**Key notes**

- This nationwide investigation demonstrates that paediatric *off-label* drug use is common at Swedish hospitals.
- Nearly half of all prescriptions were not documented for use in children.
- The findings emphasize a great need for paediatric clinical studies as well as compilation of existing clinical experience and scattered evidence.

Use of *off-label* or unlicensed drugs has been associated with a potentially increased risk of adverse drug events and other drug-related problems (3,4,8,9). The magnitude of this issue has yet to be investigated.

As a consequence of the previous lack of paediatric data on medicinal products, a new legislation was introduced into the European Union (EU) in 2007 (10). One of the tasks within the Paediatric Regulation was to collect data on use of medicines among children in all EU member states, to assess the current situation and to demonstrate within which therapeutical areas there is an unmet need for additional paediatric studies and also to create a baseline for future comparison. Therefore, in response to the Paediatric Regulation, this nationwide cross-sectional prospective study was performed at Swedish hospitals to investigate the use of drugs among children aged 0 to <18 years.

The aim of the current study was to collect comprehensive and detailed information on prescribed drugs in order to estimate the use of *off-label* and unlicensed as well as extemporaneously prepared drugs (EPD) among hospital-treated paediatric patients.

## Patients and Methods

### Data collection

All paediatric hospitals in Sweden (n = 34) received written and oral information about the design and purpose of the study at several meetings, and special prescription forms were distributed by mail prior to the study periods. In addition, some non-paediatric hospitals (n = 7) with departments that treat children also volunteered to participate, resulting in 41 hospitals finally being enrolled in this study. The majority of participating hospitals were general paediatric departments including neonatal and emergency paediatric clinics. All university hospital departments (n = 7), offering even more specialized care (e.g. paediatric /neonatal intensive care, oncology, paediatric surgery), did also participate in the study.

Data on all issued drug prescriptions to the paediatric patients in hospital care were collected during 48 h in May and October, 2008, respectively, by caring nurses and physicians. The prescription form requested information about patient social security number, age, gender, weight and cause of hospital admittance, as well as name of the drugs, indication, strength, dosage, form and route of administration, and estimated duration of drug treatment.

Patient social security numbers, which are unique to each individual, were collected to identify the exact number of patients and prevent duplication registration and were deleted before analysis. A few patients who had received drug treatment at both occasions were only recorded once, and use of same drug at both occasions was only recorded once. However, if a unique drug was only administered at one of the two occasions, this drug was recorded for that patient. Thus, all drugs administered to these patients, regardless of timeperiod, were documented for each individual.

**Figure 1:**
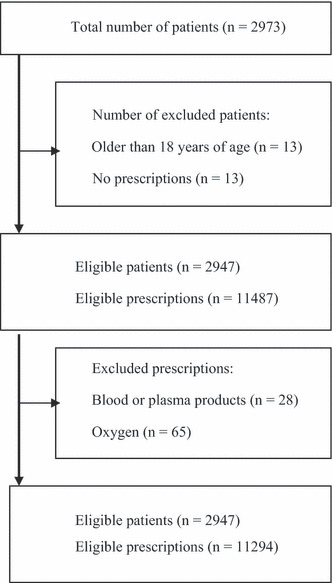
Flowchart over all paediatric patients for whom a prescription form was completed.

Only patients <18 years of age who received any drug treatment were included in the final analysis (Fig. 1). Data received concerning prescriptions of blood products or oxygen were excluded from further analysis (Fig. 1). Data on patients who did not receive any drug treatment were not collected.

### Classifications

The patients were divided into four different age groups: neonates (0–28 days), infants (>28 days <24 months), children (2 < 12 years) and adolescents (12 < 18 years) (11).

The authorization status of any given drug was defined according to the Summary of Product Characteristics (SmPC) found in the Swedish catalogue of approved medicinal products (FASS) (12) or at the Medical Products Agency homepage (13). All drugs that were listed in the SmPC in one of the two reference sources (12,13) were regarded as authorized. The term ‘unlicensed’ was applied to drugs that were not currently authorized in Sweden. Drugs prepared at pharmacies were defined as EPD. All authorized drugs were classified according to the anatomical therapeutic chemical (ATC) classification to the fifth level (14).

The *off-label* assessment was performed on all prescriptions of authorized drugs by analysing the SmPC information available in 2008. Sections 4.1, 4.2, 4.3 and 4.4 of the SmPC, which concern indications, posology and method of administration, contraindications and special warnings and precautions for use, were used to assess the *off-label* status of prescribed authorized drugs. Also, a search for the terms ‘child/children’ was performed on the complete SmPC text.

Within this study, the term *off-label* was defined as any drug use outside the terms of the SmPC. *Off-label* prescriptions were further divided into seven different categories (I–VII): A prescribed drug was considered to be *off-label* with respect to age (I) or weight (II) if the drug was explicitly not recommended for a certain age group or for children below a certain weight. Use of drugs with complete absence of paediatric information in the SmPC (III) or a stated lack of clinical data among paediatric patients (IV) was classified as *off-label* for all paediatric patients <16 years of age. Authorized drugs that, according to the SmPC, were contraindicated (V) in children were also classified as *off-label*. Drugs prescribed for indications (VI) not listed in the SmPC as well as drug prescriptions administered (VII) by a route not approved according to the SmPC were regarded as *off-label*.

Product information allowing paediatric use in general, without any age or dose specification, rendered the prescribed drug an *off-label* status if given to patients <1 year of age (I). A single prescription could be regarded as *off-label* in more than one category.

### Data analysis

Data were entered and analysed in a database (Microsoft Access 2000). All data were connected to a file with all authorized drugs and the most common unlicenced drugs and EPDs used in the paediatric population. These data were linked to the ATC classification system (14). Information concerning treatment indication in the prescription form was translated into the international classification of disease system, ICD10, by the first letter (15). Indications related to anaesthesia and/or surgery in general were coded as separate categories, as were diagnostics. If the indication was lacking for a drug prescription, it was regarded as unspecified. Data lacking in other fields, such as route of adminstration, formulation, duration of treatment and cause of treatment, were classified as unknown, unless it was clearly described elsewhere in the prescription form.

The *off-label* analysis was performed for each prescription of an authorized drug. Unlicensed drugs and EPDs were not included in the *off-label* assessment. The *off-label* assessment was validated through an independent analysis of a random sample of 20% of the different pharmaceutical compounds by a hospital pharmacist and was found to be in accordance with the initial *off-label* analysis. Statistical analysis was performed in Microsoft Excel. The study was approved by the Ethics Committee at Karolinska Insitutet, Stockholm (Dnr 2008/503-31/2).

## Results

### All prescriptions

**Figure 2:**
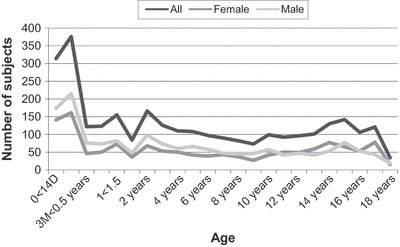
Age and gender distribution among study subjects.

Data from 41 hospitals were received, comprising a total of 2947 paediatric patients (Table 1). The median age of the study population was 4 years, with 54% boys. More than one-third of the patients were *neonates* and *infants* with a higher representation of boys (Fig. 2).

**Table 1:** Percentages of prescriptions of authorized drugs, drugs used *off-label* (A), unlicensed drugs (B), extemporaneously prepared drugs (EPD; C) and the sum of ‘undocumented’ drug use (A, B, C; *off-label,* EPD and unlicensed drugs) by age groups

Age groups	Patients	All prescriptions	Authorized drugs N (%)	A *Off-label*N (%)	B Unlicensed N (%)	C EPD N (%)	Sum of A, B, C N (%)
All	2947	11 294	9524 (84)	3879 (34)	514 (4.6)	1126 (10)	5519 (49)
Neonates	476	1875	1280 (68)	729 (57)	139 (7.4)	419 (22)	1287 (69)
Infants	698	2644	2144 (81)	1021 (48)	130 (5.0)	312 (12)	1463 (55)
Children	1043	3800	3392 (89)	1380 (41)	130 (3.4)	260 (6.8)	1770 (47)
Adolescents	730	2975	2708 (91)	749 (28)	115 (3.9)	135 (4.5)	999 (34)

All patients treated in hospital are reported at discharge to the Swedish National Board of Health and Welfare Discharge Register (16). According to data retrieved from this in-hospital patient register, a total of 3685 paediatric patients were admitted at Swedish hospitals within the study periods, with 54% boys and a median age of 6 years. Thus, our sample constituted more than 70% of all children in hospital care during the study periods.

The study population had been given 11 294 prescriptions of 948 different drugs and 37 different nutritional or technical products, with a median of three prescriptions per patient, ranging from one to 35. The majority of the prescriptions consisted of authorized drugs (Table 1). Nutritional and technical products accounted for 1% of all prescriptions. The drug prescriptions consisted of 744 authorized drugs, 120 EPDs and 84 unlicensed drugs resulting in 502 different generic substances. Paracetamol, carbohydrates, electrolytes and morphine were the most commonly prescribed substances (Table S1 in Supporting Information).

Treatment indication was known for 89% (n = 10 049) of the prescriptions. The most common treatment indication was pain (n = 1915), followed by indications associated with infection (n = 1144), prematurity (n = 981), nutrition difficulties (n = 702) and surgery or anaesthesia (n = 685). Fifty-six per cent of the prescriptions were used for treatment, 34% for prevention, 4.3% for diagnostic purposes and the remaining prescriptions (n = 568) for combinations of treatment, prevention and/or diagnostic purposes.

Duration of treatment was known for 88% (n = 9912) of all the prescriptions. A longer duration of treatment (>1 week) was reported in 43%, and duration of treatment for <1 week was reported for 22% of the prescriptions. The remaining prescriptions concerned single doses or on demand drugs (35%). Infants and neonates received more than half (53%) of all prescriptions for a longer duration than 1 week, whereas children and adolescents received 74% of all prescriptions as a single dose or on demand medication.

Formulation and route of administration were known for more than 98% (n = 11 082) of the prescriptions. Forty per cent of all prescriptions were oral (tablets, oral solutions), 35% were intravenous, and the remaining 25% were rectal, inhalation, topical or other formulations. Five per cent (n = 531) of all the prescriptions were administered in a manner not in agreement with the intended route of administration. The most frequent aberrant route of administration was the use of an intravenous drug formulation (glucose infusion) for oral administration for pain relief.

**Figure 3:**
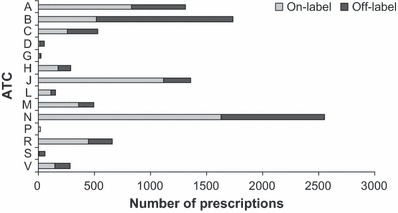
Distribution of prescriptions of authorized drugs (N = 9524) and *off-label* drug prescriptions (N = 3879) for the main anatomical therapeutic chemical classification index (ATC) subgroups. A, Alimentary tract and metabolism; B, Blood and blood-forming organs; C, Cardiovascular system; D, Dermatologicals; G, Genito-urinary system and sex hormones; H, Systemic hormonal preparation excluding sex hormones and insulins; J, Antiinfectives for systemic use; L, Antineoplastic and immunomodulating agents; M, Musculo-skeletal system; N, Nervous system; P, Antiparasitic products, insecticides and repellents; R, Respiratory system; S, Sensory organs; V, Various.

The most frequently prescribed authorized drugs classified according to ATC-codes were drugs for the nervous system (N), for example, paracetamol, drugs for blood or blood-forming organs (B), for example, glucose infusions, and drugs for infections (J), for example, bensylpenicilline (Fig. 3).

Unlicensed drugs, as well as EPDs, were mostly given by the oral route, 56% and 42%, respectively. The highest proportion of EPD prescriptions were intravenous morphine, followed by intravenous caffeine and oral salt formulations (Table 2). The most common unlicensed drugs were multivitamins and allergen extracts (Table 3). The highest percentage of use of EPD or unlicensed drugs was found among neonates and infants (Table 1).

**Table 2:** The most commonly prescribed extemporaneously prepared drugs (EPD; N = 1126)

EPD	N (%)
Morphine (intravenous)	181 (16)
Caffeine citrate (oral solution)	86 (7.6)
Phosphate (oral solution)	68 (6.0)
Calcium (oral solution)	63 (5.6)
Folic acid (oral solution)	60 (5.3)
Ergocalcipherole (oral solution)	56 (5.0)
Midazolam (oral solution)	45 (4.0)
Glucose (intravenous)	40 (3.6)
Heparin intravenous)	35 (3.1)
Total parenteral nutrition (intravenous)	31 (2.7)

**Table 3:** The most commonly prescribed unlicensed drugs (N = 514)

Unlicensed drugs	N (%)
Multivitamins	214 (42)
Allergen extracts	108 (21)
Ketamine	10 (1.9)
Technetium	10 (1.9)
Clonidine	10 (1.9)
Clobazam	10 (1.9)
Promethazine	10 (1.9)
Melatonin	9 (1.8)
Ferrans fumarate	6 (1.2)
Prochlorperazine	6 (1.2)

### Off-label prescriptions

In total, 41% of all authorized drugs were given *off-label* and the highest proportion of *off-label* prescriptions occurred in *neonates* and *infants* (Table 1). At least one *off-label* drug was prescribed to 60% of the study population, whereas 17% received at least three, and 6% at least five *off-label* drug prescriptions.

*Off-label* drug prescriptions varied considerably among ATC-groups. The largest number of *off-label* drug prescriptions was found among drugs for the nervous system (N), mostly analgesics, blood and blood-forming organs (B), and the alimentary tract and metabolism (A). The highest proportion of *off-label* classification was found among drugs for the eye (S), for the skin (D) and drugs for blood or blood-forming organs (B) (Fig. 3).

Among the most common authorized substances used *off-label* were diclofenac, morphine, midazolam and epinephrine (Table 4). Absence of paediatric information (III) in the SmPC was by far the most common reason for *off-label* classification of authorized drugs in all age groups (Table S2 in Supporting Information). Age (I) and route of administration (VII) were the most common reasons for *off-label* classification among infants. A stated lack of paediatric data (IV) and indication (VI) were the most common reasons for *off-label* classification among adolescents. In children, age (I) and indication (VI) were among the most common reasons for *off-label* classification. The most commonly prescribed drugs for which there was a stated lack of paediatric data (IV) were found among drugs used for the nervous system, blood and blood-forming organs, alimentary tract and metabolism and the cardiovascular system (Table S2 in Supporting Information). The distribution of the other different *off-label* categories with regard to ATC-codes is presented in Table S2.

**Table 4:** The most commonly prescribed authorized substances used in an *off-label* manner (n = 3879)

Substances	ATC	N (%)
Carbohydrates	B	479 (12)
Electrolytes with and without carbohydrates	B	341 (8.8)
Paracetamol	N	320 (8.2)
Sodium chloride	B	113 (2.9)
Epinephrine	C	103 (2.7)
Morphine	N	102 (2.6)
Midazolam	N	87 (2.2)
Sulfamethoxazole/trimethoprim	J	84 (2.2)
Diclofenac	M	83 (2.1)
Heparin	B	81 (2.1)

## Discussion

This study is, to our knowledge, the first national population survey of paediatric drug utilization at hospital level. The strength of this study is the large population-based sample covering information on a national level. One weakness is the lack of exact information on the amount of missing data and that the study approach was descriptive and therefore did not analyse associations between *off-label* drug use and underlying conditions. Psychiatric drugs are almost lacking in this data set, probably because few paediatric psychiatric patients were actually treated as hospital in-patients. As the study did not collect information on the number of children that did not receive any drug treatment, it is not possible to calculate prevalence.

To investigate the external validity of the data regarding the number of patients, information concerning all paediatric hospital admissions during the two study periods was retrieved from the National Board of Health and Welfare (16). The study appears to have captured information from at least 70% of all admitted children during the study periods, which supports that our data adequately reflect paediatric drug use at Swedish hospitals in general (16). Therefore, we believe that the study provides a good estimate of the current drug use by children at Swedish hospitals.

In accordance with other studies, this study could confirm that children are exposed to a large proportion of drug treatment that is not authorized by regulatory authorities (4,5,17), meaning that the safety and efficacy of the treatment is neither well studied, nor sufficiently documented. Neonates, in particular, and infants have the highest use of EPDs, which generally are poorly documented, and unlicensed drugs, as well as a substantial *off-label* drug use, which is in agreement with other studies (1,4,6,17–19). It should be noted that the definition of *off-label* drug use is sometimes regarded differently by different investigators resulting in difficulties when comparing studies.

Another finding was that these young children generally received drug treatment for a longer duration. As neonates and infants are particularly vulnerable because of their small body size and immature renal and hepatic function, further evidence of both safety and efficacy of drugs is urgently needed in these age groups.

The high rate of *off-label* prescriptions indicates a lack of appropriate dosage form in relation to age and/or weight and strongly supports the need for suitable paediatric drug forms and strengths (20–22). Thus, safer use of many drugs could be achieved by providing the actual dose sizes needed for neonates, infants and children.

Medicinal products from the ATC-group ‘blood or blood-forming organs’ (B) were given to many paediatric patients with a high proportion of *off-label* classification. The major part of this *off-label* classification was fluid therapy with carbohydrates and electrolytes. Those drugs often lack paediatric information, or there is a stated lack of clinical data in the SmPC, which has also been reported by others (19,23). It could be argued that fluid therapy could be used according to clinical guidelines rather than requesting all individual products to have specific dosing information. An important reason for a considerable use of electrolyte substitution and carbohydrate products being classified as *off-label* was the fact that those products, intended for intravenous use, were used orally, presumably because of lack of authorized oral drug forms, as has also been reported elsewhere (17). This is a safety concern because there is an apparent risk of confusing the routes of administration.

Medicinal products for the nervous system (N), with paracetamol as the most commonly prescribed substance both on-label and *off-label*, were frequently prescribed, a finding that has also been reported by others (19,21,24,25). In our data, use of paracetamol was mainly classified as *off-label* for age or weight, which can be explained by unclear information in several of the SmPCs for generic products.

A substantial *off-label* drug use was also found for other analgesics, such as opioids, diclofenac and midazolam, which has been highlighted by other authors (17,22,23,26) as an area where clinical trials appear to be needed to provide well-documented doses for all age groups. As a consequence, analgesics are listed on the European Medicines Agency (27) priority list of paediatric drugs.

The finding in our study that the most common reason for *off-label* drug prescribing is a total absence of paediatric information in the SmPC, in contrast to the situation in adults, in whom *off-label* use often concerns the indication, is in agreement with other studies (28). In recent years, the Paediatric Regulation has resulted in an increase in clinical trials in children, which will provide data on efficacy and safety of many new drugs for children. As a consequence of the Paediatric Regulation, age appropriate formulations will also be available to a higher extent for new products. The use of several drugs that explicitly lack paediatric data also underlines the need for SmPC update for many old products, for which data may be available (29). The Paediatric Regulation requests that paediatric studies on old products be submitted to competent authorities for assessment, which may result in an update of the SmPC. This is, however, going to be a time-consuming activity as there are around 1000 such old products on the market in Europe. Several important areas of high *off-label* drug use of old medicines will probably remain as there is no legal pressure and limited financial incentive to perform clinical studies in children.

A few studies have shown that *off-label* drug use is more often associated with paediatric adverse drug reactions (ADR) than on-label use (8,9,30–32). For EPD and unlicensed drugs, there is currently no clear process for collecting information on ADRs as there is for the monitoring of pharmacovigilance of authorized drugs. Therefore, spontaneous reporting of ADRs remains an important tool to discover hazardous effects, even though it is well known that the spontaneous reporting system is subject to substantial under-reporting (33,34).

## Conclusions

Paediatric *off-label* drug use is common at Swedish hospitals and nearly half of all prescriptions are not documented for use in children. This apparent lack of evidence regarding many medicinal products prescribed to children may have both efficacy and safety implications for everyday routine use of drugs. The findings emphasize a great need for paediatric clinical studies as well as compilation of existing clinical experience and scattered evidence, particularly for drug treatment in infants and neonates.

## SUPPORTING INFORMATION

**Table S1** Most commonly prescribed drug substances.

**Table S2** *Off label* prescribing ofauthorized drugs (n = 3879) and the reason for *off label* classification in each age group and by ATC code.
