# Supplementary material for: Paediatric drug use with focus on off-label prescriptions at Swedish hospitals – a nationwide study
Source: Acta Paediatr. 2012 Jul;101(7):772–8. doi: 10.1111/j.1651-2227.2012.02656.x (PMC3437470; doi:10.1111/j.1651-2227.2012.02656.x)
Supplement: Supplementary file 1 [file apa0101-0772-SD1.doc]

**Table S1**. Most commonly prescribed drug substances.

| **Drug substance** | **N (%)** |
| --- | --- |
| Paracetamol | 1230 (11) |
| Carbohydrates | 561 (5) |
| Electrolytes | 303 (3) |
| Morphine | 301 (3) |
| Ibuprofen | 256 (2) |
| Furosemide | 248 (2) |
| Salbutamole | 243 (2) |
| Multivitamins | 216 (2) |
| Midazolam | 199 (2) |
| Diclofenac | 166 (1) |

**Table S2**. *Off label* prescribing of authorized drugs (n=3879) and the reason for *off label* classification in each age group and by ATC code. One prescription can be classified as *off-label* within several categories.

|  | ***All***  ***Reasons (I-VII)*** | ***Age (I)*** | ***Weight (II)*** | ***Absence of information (III)*** | ***Stated lack of clinical data (IV)*** | ***Contra-indication (V)*** | ***Indication (VI)*** | | ***Admini-stration (VII)*** |
| --- | --- | --- | --- | --- | --- | --- | --- | --- | --- |
| ***All***  ***patients*** | ***4782*** | ***831*** | ***282*** | ***1878*** | ***419*** | ***219*** | ***665*** | ***488*** | |
| ***Neonates*** | 953 | 252 | 30 | 376 | 30 | 22 | 120 | 123 | |
| ***Infants*** | 1423 | 307 | 143 | 426 | 75 | 59 | 193 | 220 | |
| ***Children*** | 1586 | 223 | 99 | 694 | 162 | 105 | 196 | 107 | |
| ***Adolescents*** | 820 | 49 | 10 | 382 | 152 | 33 | 156 | 38 | |
| **A. Alimentary tract & metabolism** | 532 | 118 | 18 | 216 | 81 | 3 | 70 | 26 | |
| **B. Blood & blood forming organs** | 1610 | 39 | 52 | 874 | 129 | 87 | 179 | 250 | |
| **C. Cardiovascular system** | 423 | 84 | 1 | 135 | 41 | 0 | 66 | 96 | |
| **D. Dermatologicals** | 48 | 9 | 0 | 29 | 4 | 0 | 5 | 1 | |
| **G. Genito-urinary system & sex hormones** | 14 | 1 | 0 | 7 | 3 | 0 | 2 | 1 | |
| **H. Systemic hormonal preparation excl sex hormones and insulins** | 120 | 14 | 0 | 66 | 2 | 1 | 34 | 3 | |
| **J. Antiinfectives for systemic use** | 246 | 68 | 2 | 6 | 20 | 108 | 37 | 5 | |
| **L. Antineoplastic & immunomodulating agents** | 47 | 11 | 0 | 18 | 12 | 0 | 6 | 0 | |
| **M. Musculo-skeletal system** | 169 | 32 | 10 | 73 | 12 | 0 | 41 | 1 | |
| **N. Nervous system** | 1115 | 377 | 198 | 255 | 106 | 10 | 87 | 82 | |
| **P. Antiparasitic products, insecticides & repellents** | 1 | 0 | 0 | 1 | 0 | 0 | 0 | 0 | |
| **R. Respiratory system** | 259 | 75 | 1 | 33 | 0 | 2 | 134 | 14 | |
| **S. Sensory organs** | 58 | 3 | 0 | 43 | 8 | 0 | 4 | 0 | |
| **V. Various** | 140 | 0 | 0 | 122 | 1 | 8 | 0 | 9 | |
